# Is neuroticism relevant for old cancer survivors? A controlled, population-based study (the Norwegian HUNT-3 survey)

**DOI:** 10.1007/s00520-020-05870-7

**Published:** 2020-11-10

**Authors:** Ellen Karine Grov, Alv A. Dahl

**Affiliations:** 1 Department of Health Sciences, Oslo Metropolitan University, POBox 4, St.Olavs plass, 0130 Oslo, Norway; 2 National Advisory Unit for Late Effects after Cancer Therapy, Oslo University Hospital, Radiumhospitalet, Oslo, Norway; 3 Faculty of Medicine, University of Oslo, Oslo, Norway

**Keywords:** Neuroticism, Cancer survivors, Cancer-free controls, Old, Health consequences

## Abstract

**Purpose:**

Personality traits, particularly neuroticism, have an impact on people’s health and lifestyle. Due to lack of previous studies, we examined old cancer survivors (OCSs) versus cancer-free age-matched controls aged ≥ 70 years, regarding prevalence of high neuroticism, health problems in those with high and low neuroticism, and sociodemographic and clinical variables that were significantly associated with high neuroticism.

**Methods:**

We merged data from a Norwegian population–based health study (the HUNT-3) and from the Cancer Registry of Norway identifying OCSs. Three cancer-free controls were drawn at random for each OCS. Neuroticism was self-rated on a brief version of Eysenck Personality Questionnaire. Between-group statistical comparisons were made between OCS and controls, and among their subgroups with high and low neuroticism. Logistic regression analyses were used to investigate independent variables significantly associated with high neuroticism.

**Results:**

Twenty-nine percent of OCSs reported high neuroticism while controls reported 30%. OCSs showed significantly lower rate of good life satisfaction than controls. All other between-group comparisons were nonsignificant. Being OCSs was not significantly related to high neuroticism in the regression analyses. Sociodemographic, general health, and lifestyle issues, lack of energy, and low life satisfaction remained significantly associated with high neuroticism in the multivariable analysis.

**Conclusions:**

The prevalence of high neuroticism was similar in OCSs and controls. High neuroticism was associated with negative health and lifestyle issues in both groups.

**Supplementary Information:**

The online version contains supplementary material available at 10.1007/s00520-020-05870-7.

## Introduction

Cancer epidemiology shows that the number of old cancer survivors (≥ 70 years) (OCSs) is increasing [5, 34]. Compared to age-matched controls without cancer, OCSs report more health problems, reduced health-related quality of life (HRQoL), and more functional impairment [6, 24, 32, 33]. Previously we have studied how OCSs deal with activities of daily living (ADL-problems) and eventual consequences of cancer and its treatment [19]. Problems with instrumental ADL are significantly more common in OCS compared with controls (28.5% versus 21.4%). Additionally, when comparing OCS and controls, for female, those in paired relationship, reporting poor self-rated health, hospitalization last year, and low level of neuroticism, were associated with being OCS.

Several studies have examined particular problems in OCSs, such as physical symptoms, sexual and body concerns [29], balance problems, and risk of falls [22, 23]. OCSs report the latter mentioned symptoms, concerns, and risk factors as challenging. Of note are the co-occurring symptoms as pain, fatigue, and anxiety experienced by home-dwelling OCSs [36]. One study reports higher symptom burden for OCSs with progressive decline in attentional function [38]. Ahles and Root [1] emphasize that attentional processes might explain self-reported memory deficits in OCS compared with control groups’ performance on neuropsychological memory tests. In their review, the authors highlight that persistent cognitive change can be present in vulnerable subgroups of OCSs (e.g., those affected by DNA-damage or impaired hormone level) up to 20 years after facing cancer. Additionally, the aging process might exceed with cancer and its treatment; however, the mechanisms are not fully understood. Ahles and Root [1] do not include personality or neuroticism in their review. However, higher prevalence of perceived stress and depressive symptoms have been documented in the same number of OCSs and cancer-free controls (*n* = 3133) [20]. Nevertheless, the role of personality for these problems among OCSs has hardly been investigated.

In the human psyche, personality is a major factor defined as “enduring patterns of perceiving, relating to, and thinking about the environment and oneself” [2]. Personality traits are prominent aspects of personality that are exhibited in relatively consistent ways across time and situations [2]. Modern personality theory defines five basic personality traits (“The Big Five”) [27], and neuroticism is the most important one concerning health and disease [26]. “Neuroticism is the propensity to experience negative emotions, including anxiety, fear sadness, anger, guilt, disgust, irritability, loneliness, worry, self-consciousness, dissatisfaction, hostility, embarrassment, reduced self-confidence, and feelings of vulnerability, in reaction to various types of stress.” [31] [p. 2883]. High neuroticism score predisposes to many somatic diseases, mental disorders, and premature death in general but not cancer-related death [9, 26]. Additionally, high neuroticism is significantly associated with adverse effects in cancer survivors [17].

Deimling et al. [12] found that neuroticism was the strongest predictor of cancer-related worry and depression in a sample of 275 cancer survivors ≥ 60 years. The positive association between neuroticism and depression was confirmed by Chow et al. [8] among 707 cancer survivors with mean age of 71 (SD 9.1) years.

Checking several databases, we have not found any more studies examining the impact of neuroticism on health problems among OCSs. However, recent studies have documented that basic personality traits can be modified by severe traumatic life events like rape, robbery, or cancer [4, 21]. According to these findings, OCSs could be expected to have higher prevalence of high neuroticism than cancer-free controls. On this background, we used data from a Norwegian population–based health study (the HUNT-3) that had been checked for cancer in the Cancer Registry of Norway (CRN), to pose three research questions (RQs): (1) Is the prevalence of high neuroticism higher in OCSs than in cancer-free controls? (2) Are more health problems significantly associated with high neuroticism among OCSs compared to controls? (3) What independent variables are significantly associated with high neuroticism in logistic regression analyses? Our hypotheses were increased prevalence of high neuroticism in OCSs due to the cancer trauma (RQ 1), and significantly more health problems associated with high neuroticism in OCSs replicating and expanding previous findings (RQ 2). For RQ 3, we had no hypothesis since studies have shown that many variables were significantly associated with high neuroticism.

## Material and methods

### Design and sampling

The Nord-Trøndelag Health Studies (the HUNT study) (https://www.ntnu.edu/hunt) collected individual population-based data through three waves: HUNT-1 (1984-1986), HUNT-2 (1995-1997), and HUNT-3 (2006-2008). The design was identical for these waves: all inhabitants aged ≥ 20 years were invited to the survey consisting of completing questionnaires and anthropometric examination with blood sampling taking place locally in the 24 municipalities of the county. Besides recording of basic socio-demographic data (age, education, civil status), these studies covered common health problems. The HUNT findings are considered to be representative of the health problems of the total adult population of Norway [25].

**Fig. 1:**
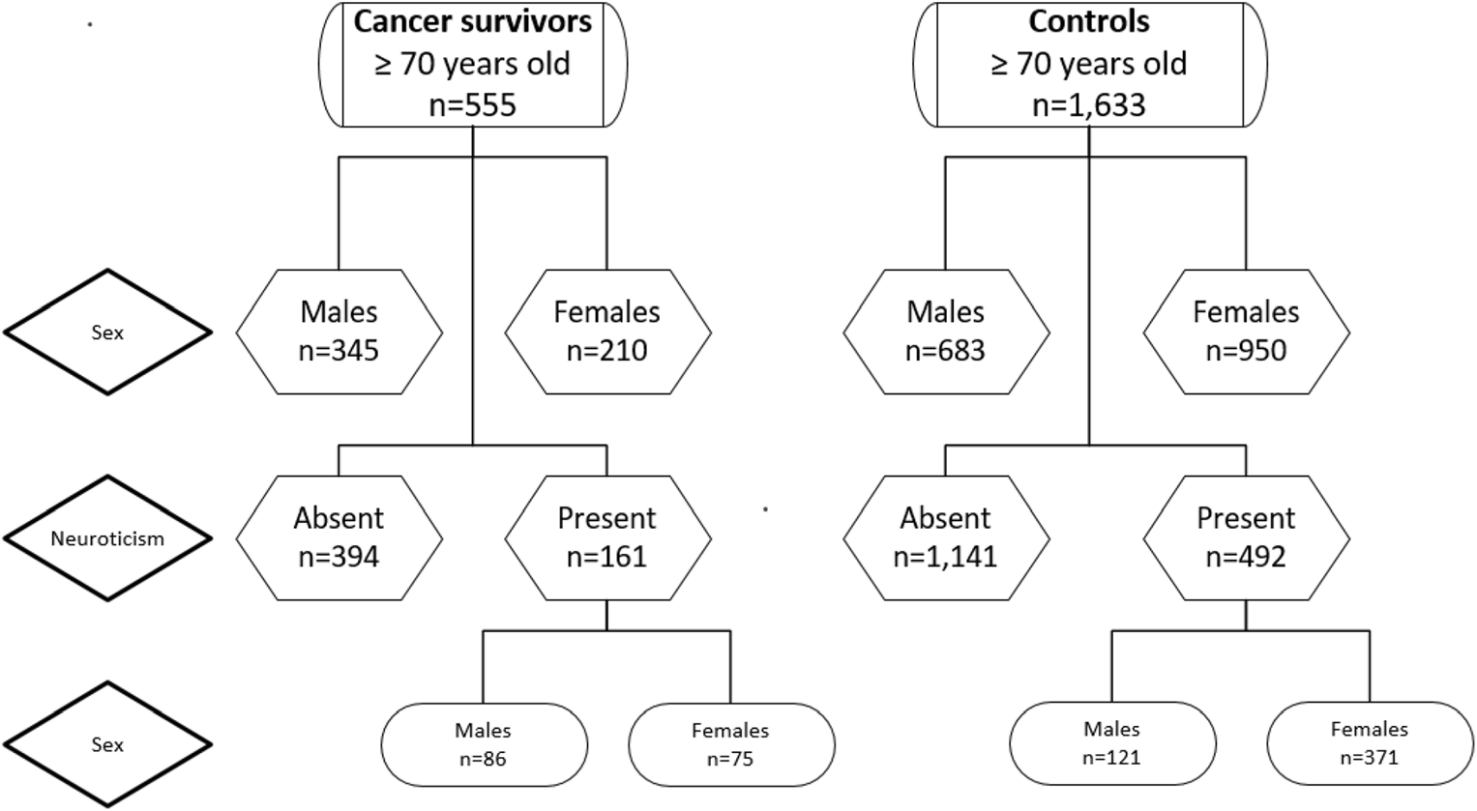
Flow chart of the sample and subsamples

Due to the unique identification number given to all persons living in Norway, participants of the HUNT surveys can be linked to The Cancer Registry of Norway (CRN). By law, the CRN contains information for all cases of cancer identified among Norwegians since 1953 (http://www.kreftregisteret.no/en/). For this study, we selected 599 OCSs with only one diagnosis of invasive cancer before their participation in the HUNT-3. The CRN identified 654 cases, but we excluded 55 participants with baso-cellular skin cancers. Among the 10,881 HUNT-3 participants not identified in the CRN (without cancer), we at random identified three controls for each OCS (*N* = 1797). The neuroticism scale was not completed by 44 persons among the OCSs and 164 among the controls (*p* = 0.19). Our study was therefore based on 555 OCSs and 1633 controls (Fig. 1).

### Measures

Neuroticism was self-rated by an abridged version of The Eysenck Personality Questionnaire (EPQ) with six items covering long-term personality characteristics (Table 1). The shortening from originally 30 items was made statistically with factor analyses identifying the six items with highest correlations with sum score of the 30-item version [37]. Each item was rated as present (1) or absent (0). The procedure was done by the late professor Kristian Tambs on data from the Norwegian Twin Registry in close collaboration with professor Hans Eysenck. The procedure has only been described in a brief internal report in Norwegian. The sum score ranged from zero to six, and was dichotomized into the high (sum scores 3–6) and low neuroticism (sum scores 0–2) group according to an established HUNT-3 procedure [16]. The Cronbach’s alpha was 0.74 in the total sample.

**Table 1:** Characteristics of old cancer survivors and cancer-free controls

Variables	Total (*N* = 2188)	Old cancer survivors (*N* = 555)	Cancer-free controls (*N* = 1633)	*p* value
Socio-demographic				
Age at HUNT-3, mean (SD)	77.1 (5.1)	77.6 (5.1)	77.0 (5.0)	0.010
Sex, *N* (%)				< 0.001
Females	1161 (53)	210 (38)	950 (58)	
Males	1028 (47)	345 (62)	683 (42)	
Relationship status, *N* (%)				0.003
Paired	1379 (65)	379 (70)	1000 (63)	
Nonpaired	749 (35)	162 (30)	587 (37)	
Education, *N* (%)				0.84*
Long (> 12 years)	298 (14)	76 (15)	222 (14)	
Short (≤ 12 years)	1759 (86)	439 (85)	1320 (86)	
Types of cancer, *N* (%)				
Prostate		171 (31)	-	
Gastrointestinal		113 (20)	-	
Breast, gynecological		85 (15)	-	
Other cancer types		187 (34)	-	
General health issues, *N* (%)				
Somatic comorbidity				0.14*
None	700 (32)	185 (33)	515 (31)	
1–2 disease(s)	1162 (52)	272 (49)	874 (54)	
3 or more diseases	343 (16)	98 (18)	245 (15)	
Poor self-rated health	901 (43)	263 (50)	638 (41)	< 0.001*
Visual and hearing problems	578 (26)	163 (29)	415 (25)	0.07*
Falls implying health care	151 (7)	42 (8)	109 (7)	0.47*
Involuntary leakage of urine	421 (22)	102 (21)	319 (23)	0.36*
Mental health issues, *N* (%)				
Memory problems	1280 (61)	333 (63)	947 (61)	0.47*
Insomnia	421 (19)	105 (19)	316 (19)	0.83*
Anxiety cases (HADS-A ≥ 8)	244 (11)	54 (10)	190 (12)	0.21*
Depression cases (HADS-D ≥ 8)	302 (14)	81 (15)	221 (14)	0.54*
High neuroticism	653 (29)	161 (29)	492 (30)	0.62*
Lifestyle issues, *N* (%)				
Daily smoker	367 (18)	97 (19)	270 (18)	0.56*
Obesity (BMI ≥ 30)	537 (35)	120 (22)	427 (26)	0.07*
≥ 5 alcoholic beverages at a time	124 (7)	28 (6)	96 (7)	0.42*
Physical activity				0.61*
Moderate (≥ 1–2 h/week)	1536 (70)	400 (72)	1138 (70)	
Hard (≥ 1–2 h/ week)	352 (30)	94 (15)	268 (16)	

*Adjusted for age at survey, sex, and relationship status

#### The Hospital Anxiety and Depression Scale

The Hospital Anxiety and Depression Scale (HADS) comprised 7 items each on the anxiety and depression subscales rated for last week. The item scores ranged from 0 (“not present”) to 3 (“highly present”), so the subscale scores range from 0 (low) to 21 (high). The cut-off score for case-ness on both subscales was a score of ≥ 8. Cronbach’s alpha was 0.81 for anxiety and 0.70 for depression in the total sample [3].

#### Personal (P-ADL) and Instrumental (I-ADL) activities of daily living problems

The P-ADL and I-ADL consist of seven and nine problem items, respectively. P-ADL and I-ADL problems were defined as presence of one or more problems among the described activities [for details see [18]].

### Socio-demographic and health-related variables

In sex, males were reference in the analyses. Relationship status was dichotomized into persons living with a spouse or partner (reference) and those not doing so. Education level was categorized into those having short (≤ 12 years) and long education (> 12 years, reference). Comorbid somatic diseases were present if a medical doctor had diagnosed any of the following diseases: myocardial infarction, angina pectoris, other heart diseases, stroke, renal diseases, asthma, chronic obstructive lung disease, diabetes, rheumatoid arthritis, ankylosing spondylitis, osteoporosis, arthrosis, or fibromyalgia. The number of reported diseases was summarized and categorized as zero, 1–2, or 3 or more. A story of falls leading to health care service last year and involuntary leakage of urine were reported as present or absent. Self-rated health was based on responses on a four-point Likert scale ranging from “very good” to “poor,” dichotomized into good (very good/good) and poor (not so good/poor). Problems of vision or hearing were defined as present when the rating for one of these senses was “moderately or much reduced.”

Moderate (no sweat or dyspnea) and hard physical (sweat and/or dyspnea) activity was present if performed for more than 1–2 h a week. Obesity was present if the body mass index (BMI) was ≥ 30. Daily smoking concerned daily consumption of any number of cigarettes. Drinking five or more glasses of alcoholic beverages at least once a month was rated as yes or no.

Energy level had seven categories from “very strong and fit” to “very tired and exhausted,” and low energy level concerned those who were “rather tired” to “very tired.” Memory problems were based on the responses to a single question, and those responding “Yes, some” and “Yes, considerable” problems were considered positive. Insomnia represented problems during the last month of getting to sleep or waking up too early and not been able to go back to sleep. Insomnia was present if “often/almost every night” was rated on one or both items [30]. Satisfaction with life had seven categories from “very satisfied” to “very dissatisfied,” and good satisfaction with life included those from “rather satisfied” to “very satisfied.”

### Statistical analyses

Between-group differences of age, sex, and marital status were examined with *t* tests and with *χ*^2^ tests. Since we observed significant differences between OCSs and controls concerning age, sex, and marital status, all other between-group analyses were adjusted for these variables using multivariable linear or logistic regression analyses using OCSs versus controls as dependent variable. The same procedures were used when analyzing the differences between high versus low neuroticism separately in the OCSs and control groups.

The associations between independent variables and high versus low neuroticism as dependent were examined with bivariate and multivariable logistic regression analyses. The strength of associations was expressed as odds ratios (ORs) with 95% confidence intervals (95% CI). The level of significance was set at *p* < 0.05 and all tests were two-tailed. Data were analyzed by SPSS for PC, version 25 (IBM, Armonk, NY).

### Ethics

The Hospital Review Board for Cancer Research at Oslo University Hospital, The Norwegian Radiumhospital, The Norwegian Data Inspectorate, and The Regional Committee for Medical Research Ethics, Health Region South-East of Norway approved this study. All participants of the HUNT-3 gave written informed consent.

## Results

### Description of OCSs and controls

Prostate, gastrointestinal, and breast/gynecological cancer represented the major groups of malignancies, and the mean time from diagnosis to survey was 4.4 years (SD 3.1). Comparison between OCSs and controls revealed significant differences regarding age, sex, and relationship status (Table 1). After adjusted for these variables, significantly more OCSs reported poor self-rated health compared to controls (Table 1). High neuroticism was reported by 29% of OCSs and 30% of controls (*p* = 0.62 adjusted). The OCS group had significantly lower prevalence of good life satisfaction versus controls. All other between-group comparisons were nonsignificant after adjustments.

### Comparisons of high and low neuroticism groups

Among OCSs, the high neuroticism group had higher prevalence of females, survivors with poor self-rated health and who had falls implying health care compared to the low neuroticism group (Table 2). The high neuroticism group also had higher rates of insomnia, and anxiety and depression cases, I-ADL problems, and low energy level. That group also had lower rates of hard physical activity and of good life satisfaction compared to the low neuroticism group.

**Table 2:** Comparison of high and low neuroticism among old cancer survivor and old cancer-free controls in HUNT-3

Variables	Old cancer survivors			Cancer-free controls		
	High neuroticism (*N* = 161)	Low neuroticism (*N* = 394)	*p* value	High neuroticism (*N* = 492)	Low neuroticism (*N* = 1141)	*p* value
Socio-demographic						
Age at HUNT-3, mean (SD)	77.5 (4.8)	77.7 (5.2)	0.67	77.0 (5.2)	76.9 (5.0)	0.78
Sex, *N* (%)			0.007			< 0.001
Females	75 (47)	135 (34)		371 (75)	580 (51)	
Males	86 (53)	259 (66)		121 (25)	561 (49)	
Paired relationship, *N* (%)	107 (69)	272 (71)	0.74	274 (57)	726 (66)	0.002
Short education, *N* (%)	130 (89)	309 (84)	0.13*	417 (91)	903 (83)	< 0.001*
Types of cancer, *N* (%)			0.09*	–	–	–
Prostate	80 (31)	121 (31)				
Gastrointestinal	31 (19)	76 (19)				
Breast, gynecological	33 (21)	51 (13)				
Other cancer types	47 (29)	147 (37)				
General health issues, *N* (%)						
Somatic comorbidity present	112 (70)	258 (65)	0.34*	396 (81)	723 (63)	< 0.001*
Poor self-rated health	100 (69)	163 (43)	< 0.001*	275 (58)	363 (34)	< 0.001*
Visual and hearing problems	54 (33)	109 (28)	0.16*	144 (29)	271 (24)	0.019*
Falls implying health care	18 (11)	24 (6)	0.039*	48 (10)	61 (5)	0.001*
Involuntary leakage of urine	37 (23)	65 (16)	0.07*	127 (26)	192 (17)	< 0.001*
Mental health issues, *N* (%)						
Memory problems	104 (68)	230 (860)	0.10*	321 (68)	625 (57)	< 0.001*
Insomnia	44 (27)	61 (15)	0.001*	146 (30)	170 (15)	< 0.001*
Anxiety cases (HADS-A ≥ 8)	43 (27)	11 (3)	< 0.001*	168 (35)	22 (2)	< 0.001*
Depression cases (HADS-D ≥ 8)	40 (25)	41 (10)	< 0.001*	117 (24)	104 (9)	< 0.001*
Lifestyle issues, *N* (%)						
Daily smoker	25 (15)	52 (13)	0.46*	60 (12)	144 (13)	0.81*
Obesity (BMI ≥ 30)	40 (25)	80 (21)	0.25*	155 (32)	262 (23)	< 0.001*
≥ 5 alcoholic beverages at a time	11 (7)	17 (4)	0.21*	24 (5)	72 (6)	0.26*
Physical activity						
Moderate (≥ 1–2 h/week)	112 (70)	289 (73)	0.39*	296 (60)	841 (74)	< 0.001*
Hard (≥ 1–2 h/ week)	15 (9)	69 (17)	0.015*	50 (10)	218 (19)	< 0.001*
ADL, energy, satisfaction, *N* (%)						
I-ADL problems	56 (35)	104 (26)	0.046*	144 (29)	207 (18)	< 0.001*
P-ADL problems	5 (3)	12 (3)	1.00*	19 (4)	27 (2)	0.09*
Low energy level	42 (27)	27 (7)	< 0.001*	93 (19)	65 (6)	< 0.001*
Good life satisfaction	122 (80)	357 (92)	< 0.001*	400 (84)	1059 (95)	< 0.001*

*Adjusted for sex and paired relationship

All these significant between-group differences were also observed among controls. In addition, more controls with high neuroticism had short education and were in nonpaired relationships than controls with low neuroticism. Controls with high neuroticism also had significantly more somatic comorbidity, visual and hearing problems, memory problems, obesity, and less moderate physical activity (Table 2).

### Logistic regression analyses

We tested various independent variables versus high neuroticism (reference low neuroticism) as dependent variable in bivariate and multivariable logistic regression analyses. Anxiety and depression cases were omitted due to high correlations with neuroticism. OCSs versus controls was nonsignificant in bivariate analysis (Table 3).

**Table 3:** Bivariate and multivariable logistic regression analyses with the high neuroticism group as dependent variables (low neuroticism as reference)

Independent variables	Bivariate analyses			Multivariable analysis		
	OR	95% CI	*p*	OR	95% CI	*p*
Old cancer survivors versus cancer-free controls (reference)	0.95	0.77–1.17	0.60	0.93	0.71–1.22	0.60
Socio-demographic						
Age at HUNT-3	1.00	0.98–1.02	0.98			
Females (males reference)	2.47	2.04–3.00	< 0.001	0.41	0.32–0.53	< 0.001
Nonpaired relationship	1.34	1.10–1.62	0.003	0.85	0.66–1.08	0.18
Short education	1.95	1.43–2.65	< 0.001	1.52	1.07–2.16	0.019
General health issues						
Somatic comorbidity present	1.98	1.60–2.45	< 0.001	1.55	1.19–2.02	0.001
Poor self-rated health	2.73	2.25–3.31	< 0.001	1.87	1.47–2.39	< 0.001
Visual and hearing problems	1.32	1.08–1.62	0.007	0.98	0.76–1.26	0.87
Falls implying health care	1.92	1.37–2.69	< 0.001	1.67	1.11–2.52	0.014
Involuntary leakage of urine	1.67	1.34–2.08	< 0.001	1.15	0.88–1.52	0.30
Lifestyle issues						
Daily smoker	1.02	0.78–1.34	0.87			
Obesity (BMI ≥ 30)	1.49	1.21–1.83	< *0.001*	1.28	0.99–1.64	0.054
≥ 5 alcoholic beverages	0.92	0.62–1.38	0.70			
Physical activity			< 0.001			0.004
Hard (reference)	1.00	–		1.00	–	
Moderate	1.69	1.26–2.27	0.001	1.19	0.85–1.67	0.31
None	3.32	2.41–4.56	< 0.001	1.77	1.20–2.60	0.004
ADL, energy, satisfaction						
I-ADL problems	1.74	1.41–2.14	< 0.001	1.06	0.80–1.40	0.69
P-ADL problems	1.47	0.87–2.46	0.15			
Low energy level	4.13	3.11–5.48	< 0.001	2.11	1.48–2.99	< 0.001
Poor life satisfaction	3.49	2.58–4.72	< 0.001	2.69	1.86–3.89	< 0.001

Independent variables significantly associated with high neuroticism in bivariate analyses were entered into the multivariable analysis. In that analysis female sex, short education, somatic comorbidity, poor self-rated health, falls implying health care, no physical activity, low energy level, and poor life satisfaction remained significantly associated with high neuroticism.

## Discussion

### Answers to the research questions

As to RQ 1, we found that the prevalence of high neuroticism among OCSs was 29% and 30% among controls. Therefore, our hypothesis of higher prevalence in OCSs was not supported. Concerning RQ 2, we found more health problems between the high and low neuroticism groups among controls, which was in opposition to our hypothesis. For RQ 3, OCSs versus controls were not significantly associated with high neuroticism in the regression analyses. However, socio-demographic, general health issues, lifestyle issues, energy, and life satisfaction remained significantly associated with high neuroticism in the multivariable analysis.

### Interpretation of main findings

The stability of basic personality traits over the life cycle, and their changes due to negative life events like cancer, has been under discussion [4, 8, 21]. We found no increased prevalence of neuroticism in OCSs compared to controls. This finding indicates that cancer and its treatment and later complications did not increase the prevalence of neuroticism. A report stated that OCSs must cope with changes that accompany normal aging, but in addition they have increased risk of comorbidity, and late adverse effects [5]. In contrast, we found that few health problems were worse in OCSs compared to controls, not supporting this American statement. In contrast, we observed considerably more health problems in controls with high neuroticism than among corresponding OCSs. They concerned somatic comorbidity, visual and hearing problems, memory problems, obesity, and less moderate physical activity.

We have several explanations of these interesting and contra-intuitive between group differences. One is that the challenge of cancer strengthens coping and resilience. Another is more attention to lifestyle issue, and a third that OCSs are more aware of health problems and therefore visit their regular general practitioners more frequently, and a possible combination of these three explanations.

The results of our regression analyses mainly confirmed previous findings from general population studies of high neuroticism [26]. High neuroticism is significantly associated with more somatic comorbidity and poor self-rated health, less energy, and physical activity. The increased risk of falls leading to health care problems seems to be a new finding. It is important to state that this finding concerns older participants in general, and they are not specific for OCSs.

### Comparisons with previous studies

The main finding from previous studies among OCSs is that the prevalence of depression is significantly higher in the high versus the low neuroticism groups [8, 12]. Of note, this finding was confirmed by us in both the OCSs and the controls. However, surprisingly, OCSs are not different than old cancer-free controls regarding health and lifestyle issues. Previous studies have other research outcomes than what we have in this study, so comparisons with HRQOL and life satisfaction are difficult. We therefore recommend other researchers to include personality as a valuable variable when examining OCSs.

### New findings and their meaning

We found that anxiety disorder also was more common among OCSs with high versus low neuroticism. Falls is a common and devastating problem among old people, and its positive association with high neuroticism is a new finding. As physical symptoms and body concerns are problems for OCS [29] and neuroticism associated with mental health problems as depression [12] and cancer-related worry [12], we suggest the burden of health issues mentioned above in line with falls. Of interest is that Deimling et al. [12] found association between cancer-related worry, while we found association between falls and high neuroticism. One can reflect on any worry (cancer-related worry) and huge problems keeping on in life (falls) linked to neuroticism. Huang et al. [23] revealed dependence in ADL and presence of depression as significant independent factors associated with increased odds of reporting falls and balance/walking difficulty in the past 12 months for all cancer types; however, they did not examine personality. Corresponding to Huang et al. [23], our findings with falls implying health care, presence of somatic comorbidity, and no performance of physical activity indicate a sample of frail and fatigued OCSs, also described characteristics for this target group in previous studies [19, 36]. The high prevalence of frailty in OCSs might lead to concerns and further to their poor life satisfaction experience. Trouble sleeping is a common problem in old people, rather than more common among OCSs. Interestingly, the high neuroticism subgroups of both OCSs and cancer-free controls report significantly more insomnia problems. Neckelmann et al. [30] postulated insomnia as a general indicator for anxiety and depression. Lack of precise measures and close follow-up, not only self-reported questionnaires, might have given even more exact results regarding personality and health and lifestyle issues.

### Issues concerning neuroticism

Neuroticism as a personality trait is closely related to the emotional states of anxiety and depression, eventually as an important etiologic factor for these states [31]. High neuroticism has been documented as a risk factor of many of the diseases of Charlson’s comorbidity index either directly or through its strong association with anxiety and/or depression as etiological factors [7]. Screening for neuroticism thereby also represents a screening for the propensity for comorbid somatic diseases as well as mental disorders.

### Strength and limitations

The strengths of this study are the definite identification of OCSs and a control group without cancer, made possible by the linkage with the CRN, which combined with HUNT-3 self-report makes the definition of OCSs quite definite, without false negatives or positives. A weakness is the representativity of patients with cancer responding to the HUNT-2 and HUNT-3 as indicated from the study by Fosså et al. [14]. Particularly, there is a risk for selection bias in the way that healthier OCSs with more than 2 years since their cancer diagnosis respond to the HUNT surveys. Sensitivity analysis, including data simulation studies, on the effect of matching versus statistical adjustment is methodologically interesting, but we find such analysis outside the scope of this study. Shortening of tests like the EPQ always implies a risk of loss of validity [35]. We have not tested that risk in our study, and our findings should be considered in this regard.

### Clinical implications

If this pattern of socio-demographic impact, impaired general health issues, problematic lifestyle issues, loss of energy, and poor life satisfaction represent all OCSs, health care personnel must be aware of those with high neuroticism since they are an extremely vulnerable subgroup among OCSs. Based on the findings from this study, we recommend comprehensive geriatric assessment (i.e., easy accessible, standardized tests to evaluate frailty; physical, mental, cognitive, and social function) [13, 15, 28], to be exceeded with six questions on neuroticism suggested from The Eysenck Personality Questionnaire. As Ahles and Root emphasize, we need a multidimensional perspective to examine OCS [1]. Thus, we recommend assessment tools that cover a wide range of aspects for OCS [10, 11].

## Conclusion

From this register-data study, we suggest that high neuroticism is associated with physical as well as mental impairment and vulnerability in OCSs. Screening for physical and mental impairment by means of a short test battery, geriatric assessment, balance test, and neuroticism, might serve as mapping of OCSs’ situation and give indications for helpful interventions.

## Supplementary Information

ESM 1 (DOCX 14 kb)

## Data Availability

Data is available on request to The HUNT Research Centre; https://www.ntnu.edu/hunt.
