# Supplementary material for: Is neuroticism relevant for old cancer survivors? A controlled, population-based study (the Norwegian HUNT-3 survey)
Source: Support Care Cancer. 2020 Nov 10;29(7):3623–32. doi: 10.1007/s00520-020-05870-7 (PMC8163664; doi:10.1007/s00520-020-05870-7)
Supplement: Supplementary file 1 — (DOCX 14 kb) [file 520_2020_5870_MOESM1_ESM.docx]

**Supplement**

**Neuroticism 6-item version of the Eysenck Personality Questionnaire.**

**Instruction:** The items below concern how you usually behave, feel, or act. Please, set a ring round the number for either Yes or No for each item. Please, respond quickly and do not think too long about the meaning of each item.

| **Items** | **Yes** | **No** |
| --- | --- | --- |
| 1. Are you often worried? | 1 | 0 |
| 1. Are your feelings easily hurt? | 1 | 0 |
| 1. Do you often feel that you lose interest? | 1 | 0 |
| 1. Do you worry too long after an embarrassing experience? | 1 | 0 |
| 1. Do you often feel tired and indifferent/unmotivated without reason? | 1 | 0 |
| 1. Do you worry that terrible things might happen? | 1 | 0 |

Low neuroticism: sum score 0 – 2; High neuroticism: sum score 3 – 6 (reference Grav et al. 2012)
